# Diabetes mellitus—Definition, Klassifikation, Diagnose, Screening und Prävention (Update 2026)

**DOI:** 10.1007/s00508-025-02627-2

**Published:** 2026-04-30

**Authors:** Jürgen Harreiter, Michael Roden

**Affiliations:** 1 Abteilung für Innere Medizin mit Palliative Care, Landesklinikum Scheibbs, Scheibbs, Österreich; 2 Abteilung für Endokrinologie und Stoffwechsel, Universitätsklinik für Innere Medizin III, Medizinische Universität Wien, Wien, Österreich; 3 Klinik für Endokrinologie und Diabetologie, Medizinische Fakultät und Universitätsklinikum, Heinrich-Heine-Universität, Düsseldorf, Deutschland; 4 Institut für Klinische Diabetologie, Deutsches Diabetes-Zentrum (DDZ), Leibniz-Zentrum für Diabetesforschung, Düsseldorf, Deutschland; 5 Deutsches Zentrum für Diabetesforschung, DZD e. V., München-Neuherberg, Deutschland

**Keywords:** Hyperglykämie, Glukoseintoleranz, Abnorme Nüchternglukose, Prädiabetes, Adipositas, Hyperglycemia, Glucose intolerance, Impaired fasting glucose, Prediabetes, Obesity

## Abstract

Diabetes mellitus bezeichnet eine Gruppe von heterogenen Erkrankungen, deren gemeinsamer Befund die Erhöhung der Blutglukosekonzentration ist. Die gegenwärtige Klassifikation des Diabetes mellitus wird dargestellt, und die wesentlichen Merkmale von Typ 1, Typ 2 und der häufigsten Form des monogenetischen Diabetes (MODY) werden gegenübergestellt. Darüber hinaus werden die Kriterien für die korrekte biochemische Diagnose unter Nüchternbedingungen und im oralen Glukosetoleranztest sowie die Anwendung von Hämoglobin A_1c_ (HbA_1c_) zusammengefasst. Die zunehmende Prävalenz des Diabetes erfordert zudem gezieltes Screening zur Erkennung von Diabetes und Prädiabetes in Risikogruppen. Dies bildet die Grundlage für die frühzeitige Einleitung von Lebensstiländerung und medikamentösen Maßnahmen zur Prävention der Manifestation des Diabetes in Risikogruppen und Verzögerung der Diabetesprogression.

## Abstract (en)

Diabetes mellitus comprises of a group of heterogeneous disorders, which have an increase in blood glucose concentrations in common. The current classification for diabetes mellitus is presented and the main features of type 1, type 2 and monogenic diabetes (maturity onset diabetes of the young [MODY]) are compared. Furthermore, the criteria for the correct biochemical diagnosis during fasting and oral glucose tolerance tests as well as the use of hemoglobin A1c (HbA1c) are summarized. The increasing prevalence of diabetes requires targeted screening for detecting diabetes and prediabetes in risk groups. This forms the basis for the early initiation of lifestyle- or drug-based measures to prevent the onset of diabetes in these risk groups and to delay the progression of diabetes.

Im Jahr 2025 wurde die weltweite Prävalenz von Diabetes mellitus bei Erwachsenen zwischen dem 20. und 79. Lebensjahr auf 589 Mio., v. a. bedingt durch Typ-2-Diabetes mellitus, geschätzt [1]. Bis zum Jahr 2050 soll diese Zahl um 45 % auf 853 Mio. zunehmen, wobei dieser Anstieg in besonderem Maß strukturärmere Regionen betreffen soll (erwartete relative Steigerung: 10–142 %) [1].

In Ländern mit niedrigen und mittleren Einkommen wird zudem eine hohe Prävalenz an nicht diagnostiziertem Diabetes angenommen (46–59 %) [2]. Die in Österreich aufgrund fehlender nationaler Register geschätzte Diabetesprävalenz beträgt gemäß dem Österreichischen Diabetesbericht aus 2017 etwa 5–7 % [3]. Eine neuere populationsbezogene österreichische Kohortenstudie (LEAD study) mit mehr als 11.000 Teilnehmern im Alter von 6 bis 80 Jahren ergab eine Prävalenz von Vorstufen des Diabetes („Prädiabetes“) von 20,2 % (Männer: 23,6 %; Frauen: 17,1 %) und Diabetes von 5,4 % (Männer: 7,3 %; Frauen: 3,7 %) [4]. Da bereits Prädiabetes mit erhöhtem Risiko für vaskuläre Erkrankungen (koronare Herzkrankheit, Schlaganfall) und allgemeine Mortalität assoziiert ist, sind effiziente Strategien zu Früherkennung und Prävention von Prädiabetes und Typ-2-Diabetes erforderlich [5].

## Definition

Diabetes mellitus bezeichnet eine Gruppe von Stoffwechselerkrankungen, deren gemeinsames Merkmal die Erhöhung des Blutglukosespiegels, die Hyperglykämie, ist. Schwere Hyperglykämie führt von klassischen Symptomen wie Polyurie, Polydipsie, Müdigkeit und Leistungsabfall, unerklärbarer Gewichtsverlust über Sehstörungen und Infektanfälligkeit bis hin zu Ketoazidose oder nichtketoazidotischem, hyperosmolarem Syndrom mit Gefahr des Komas. Eine chronische Hyperglykämie bewirkt des Weiteren Störungen der Sekretion und/oder Wirkung von Insulin und ist mit Langzeitschäden und Funktionsstörungen verschiedener Gewebe und Organe (Augen, Nieren, Nerven, Herz und Blutgefäße) assoziiert.

## Klassifikation

Die Klassifikation des Diabetes mellitus erfolgt in 4 Typen [6, 7].

### Typ-1-Diabetes mellitus

Störung der Insulinsekretion durch überwiegend immunologisch vermittelte Zerstörung der pankreatischen Betazellen mit meist absolutem Insulinmangel. LADA (latenter autoimmuner Diabetes der Erwachsenen) bezeichnet einen autoimmun-bedingten Diabetes mellitus, der durch das Auftreten im Erwachsenenalter und den langsameren Verlust der Insulinsekretion gekennzeichnet ist, dem Typ-1-Diabetes zugeordnet wird und keinen eigenständigen Subtyp darstellt. Auch Checkpointinhibitor-induzierter Diabetes mit und ohne Autoantikörper wird dieser Klasse zugezählt [6, 7]. Das Vorhandensein von Diabetes-assoziierten (auch: Inselzell‑)Autoantikörpern ist ein starker Prädiktor für die Entwicklung eines Typ-1-Diabetes. Dabei scheinen Alter bei Feststellung, Titerhöhe sowie Anzahl und Spezifität der Autoantikörper mit der Progression des Typ-1-Diabetes assoziiert zu sein [6]. Die schrittweise Progression zur Manifestation des Typ-1-Diabetes wird in Stadien eingeteilt (Tab. 1; [6, 7]). Das Screening auf präsymptomatischen Typ-1-Diabetes durch Nachweis von Autoantikörpern gegen Insulin, GAD, IA‑2 oder Zinktransporter‑8 wird derzeit in Screeningprogrammen, bei klinischen Studien und Familienangehörigen von Menschen mit Typ-1-Diabetes empfohlen [6, 7].

**Tab. 1:** Klinische Charakteristika und spezifische Diagnosekriterien der Stadien des Typ-1-Diabetes. (Nach [6–8])

	Stadium 1	Stadium 2	Stadium 3
*Eigenschaften*			
	Normoglykämie	Dysglykämie	Manifeste Hyperglykämie
Klinik	Asymptomatisch	Asymptomatisch	Symptomatisch
Diagnosekriterien	≥ 2 Diabetesautoantikörper	≥ 2 Diabetesautoantikörper	≥ 2 Diabetesautoantikörper
*Glukose*			
Nüchtern	< 100 mg/dl (5,6 mmol/l)	100–125 mg/dl (5,6–6,9 mmol/l)	≥ 126 mg/dl (7,0 mmol/l)
2 h postprandial	< 140 mg/dl (7,8 mmol/l)	140–199 mg/dl (7,8–11,0 mmol/l)	≥ 200 mg/dl (11,1 mmol/l)
HbA_1c_	< 5,7 % (39 mmol/mol)	5,7–6,4 % (39–47 mmol/mol)	≥ 6,5 % (48 mmol/mol)

### Typ-2-Diabetes mellitus

Verminderung der Insulinwirkung (Insulinresistenz) mit fortschreitendem Verlust der Betazellfunktion, bei zunächst häufig relativem Insulinmangel und typischerweise Störung der glukoseabhängigen Insulinsekretion. Die Funktionsstörungen sind in unterschiedlicher Ausprägung schon lange vor der klinischen Manifestation des Diabetes allein oder im Rahmen eines metabolischen Syndroms mit erhöhtem Risiko für makrovaskuläre Folgen vorhanden. Die Tab. 2 listet Hinweise zur klinischen Differenzialdiagnose zum Typ-1-Diabetes und Maturity Onset Diabetes of the Young (MODY) auf.

**Tab. 2:** Differenzialdiagnostische Überlegungen zur Unterscheidung von Typ-1-, Typ-2-Diabetes und MODY. (Nach [7])

Kriterium^a^	Typ-1-Diabetes	Typ-2-Diabetes	MODY
Ätiologie und Pathogenese	Autoimmun Genetische Prädisposition	Genetische Prädisposition, multifaktoriell (Lebensstil, Umwelt)	Monogenetisch (Transkriptionsfaktoren, Glucokinase), autosomal-dominant
Klinik	Autoantikörper Absoluter Insulinmangel	Insulinresistenz und -sekretionsstörung bis hin zum (absoluten) Insulinmangel	variabel, je nach genetischem Defekt
Häufigkeit	5–10 % der Diabetesfälle	90–95 % der Diabetesfälle	Ca. 2 % der Diabetesfälle
Manifestationsalter	Meist in Kindheit oder Jugend (Ausnahme: LADA)	Meist im höheren Alter, zunehmend frühere Manifestation	Adoleszenz bis frühes Erwachsenenalter
Körpergewicht	Meist normalgewichtig	Meist übergewichtig, adipös	Meist normalgewichtig
Symptome	Akut: Polyurie, Polydipsie, schwere Hyperglykämie, Ketoazidose	Langsamer Beginn, häufig bereits Begleiterkrankungen zum Zeitpunkt der Diagnose, moderate Hyperglykämie	Langsam einsetzende, variable Hyperglykämie
Neigung zur diabetischen Ketoazidose (DKA)	Ja	Selten	Nein
Begleiterkrankungen	Andere Autoimmunerkrankungen (z. B.: Hashimoto-Thyreoiditis, Zöliakie)	Viszerale Adipositas, Hypertonie, Dyslipidämie, Arteriosklerose, MASLD	Nierenzysten, Pankreasatrophie, Augenbeteiligung abhängig von MODY-Typ
Plasma C‑Peptid	Meist niedrig bis fehlend	Meist normal bis erhöht	Normal bis niedrig
Insulinresistenz	Meistens nein	Meistens ja	Nein
Diabetes-assoziierte Antikörper	Ja (GAD, ICA, IA‑2, IAA, ZnT8)	Keine	Keine
HLA-Assoziation	HLA-DR/DQ	Keine	Keine
Therapie	Insulin	Lebensstilmodifikation, orale Glukosesenker, Inkretinagonisten, Insulin	Oft keine erforderlich, orale Glukosesenker, Insulin (je nach MODY-Typ)

^a^Symptome, Klinik und Verlauf der Diabetestypen weisen aber eine hohe Variabilität auf, die eine Differenzialdiagnose im Einzelfall erschweren kann. Bei unklarem Diabetestyp oder untypischer Klinik sollte immer auch an andere seltene Diabetesformen (z. B. MODY) gedacht werden

Rezente Studien schlagen eine weitere Klassifizierung des Typ-2-Diabetes in Subtypen (Endotypen) auf Basis unterschiedlicher Phänotypen [9, 10] oder auch Genotypen [11, 12] vor, die Unterschiede im Ausmaß der Insulinresistenz und Betazellfunktion sowie Risiken für Diabetes-bedingte Komplikationen aufweisen. Eine solche Klassifikation könnte zukünftig als Basis einer neuen Typisierung des Diabetes dienen und eine stratifizierte bzw. präzisere Prävention und Therapie ermöglichen. Dies erfordert allerdings weitere Studien, sodass derzeit eine solche Klassifizierung für die klinische Praxis aktuell noch nicht empfohlen werden kann.

### Andere spezifische Diabetestypen

Erkrankungen des exokrinen Pankreas (z. B. Pankreatitis, Traumen, Operationen, Tumoren, Hämochromatose, zystische Fibrose), endokriner Organe (z. B. Cushing-Syndrom, Akromegalie), medikamentös-chemisch (z. B. Glukokortikoide, α‑Interferon, Posttransplantationsdiabetes, HAART bei HIV/AIDS), genetische Defekte der Insulinsekretion (z. B. Formen des MODY) und der Insulinwirkung (z. B. lipoatropher Diabetes), andere genetische Syndrome (z. B. Down‑, Klinefelter‑, Turner-Syndrome), Infektionen (z. B. kongenitale Röteln) und seltene Formen des autoimmun-vermittelten Diabetes (z. B. „Stiff-man“-Syndrom). Details finden sich in der Leitlinie „Andere spezifische Diabetesformen und exokrine Pankreasinsuffizienz“.

### Gestationsdiabetes (GDM)

Erstmals im zweiten oder dritten Schwangerschaftstrimester aufgetretene bzw. diagnostizierte Glukosetoleranzstörung. Voraussetzung ist, dass außerhalb der Schwangerschaft kein Diabetes mellitus bestanden hat. (siehe: Leitlinie „Gestationsdiabetes“).

Aufgrund einer oft unklaren Vorgeschichte ist eine Unterscheidung zwischen den einzelnen Diabetestypen zum Zeitpunkt der Diagnosestellung vor der notwendigen ausführlichen Anamnese und dem Eintreffen aller erforderlichen Befunde nicht immer möglich.

Insulinabhängigkeit stellt keine Klassifikation dar.

## Diagnose

Die Diagnose des Diabetes erfolgt anhand von Nüchternglukose, Gelegenheitsglukose, oralem Glukosetoleranztest (OGTT) oder Hämoglobin A_1c_ (HbA_1c_). Die Hyperglykämie entwickelt sich kontinuierlich, und die Störungen von Nüchtern- und postprandialer Glykämie weisen unterschiedliche Zeitverläufe auf. Die etablierten Grenzwerte sind daher nicht in kompletter Übereinstimmung in der Identifizierung von Menschen mit Diabetes, des Weiteren unterliegen alle Tests einer Variabilität, sodass eine zeitnahe Testwiederholung oder Bestätigung eines Testresultates durch einen anderen Test – außer bei Vorliegen klassischer klinischer Symptome – immer erforderlich ist.

### Nüchternglukose, Gelegenheitsglukose und OGTT

Die Diagnose wird unabhängig von Alter und Geschlecht durch Messung mehrfach erhöhter Blutglukosewerte an mindestens 2 verschiedenen Tagen gestellt (Tab. 3). Bei klinischem Verdacht und widersprüchlichen Ergebnissen wird die Diagnose mittels OGTT gestellt. Als „normal“ gelten derzeit Nüchternglukosewerte im venösen Plasma von < 100 mg/dl (< 5,6 mmol/l) bzw. postprandiale Werte < 140 mg/dl (< 7,8 mml/l). Niedrigere Werte schließen das Vorliegen einer Glukosestoffwechselstörung oder Folgeschäden aber nicht aus. Die Grundlage für die Wahl der Grenzwerte liegt in der überwiegend kontinuierlichen Beziehung zwischen höheren Blutglukosewerten (nüchtern und 2 h nach oraler Glukosebelastung) und der Zunahme des Risikos für Folgeschäden.

**Tab. 3:** Standarddiagnostik des Diabetes mellitus und des erhöhten Diabetesrisikos

	Manifester Diabetes mellitus	Erhöhtes Diabetesrisiko (Prädiabetes)^a^
Nicht-Nüchtern, Gelegenheitsglukose („Random-Glucose“, venös oder kapillär)	≥ 200 mg/dl (11,1 mmol/l) an 2 Tagen^b^ ODER ≥ 200 mg/dl (11,1 mmol/l) + klassische Symptome^c^	–
Nüchternglukose (venöses Plasma)	≥ 126 mg/dl (7,0 mmol/l) an 2 Tagen^b^	≥ 100 mg/dl (5,6 mmol/l), aber < 126 mg/dl (7,0 mmol/l) (abnorme Nüchternglukose, „impaired fasting glucose“ [IFG])
2‑h-Glukose nach 75 g OGTT (venöses Plasma)	≥ 200 mg/dl (11,1 mmol/l) an 2 Tagen^b^	Glukose ≥ 140 mg/dl (7,8 mmol/l), aber < 200 mg/dl (11,1 mmol/l) (gestörte Glukosetoleranz, „impaired glucose tolerance“ [IGT])
HbA_1c_	≥ 6,5 % (48 mmol/mol) an 2 Tagen^b^	≥ 5,7 % (39 mmol/mol), aber < 6,5 % (48 mmol/mol)^d^

^a^Ein erhöhtes Diabetesrisiko kann auch ohne Nachweis von Störungen der Glykämie bestehen und lässt sich mittels definierter Risikotests erheben (s. unter: Prävention)

^b^Sind 2 unterschiedliche Tests positiv, ist die Diagnose Diabetes gegeben, sodass auf die Testwiederholung verzichtet werden kann. Ergeben unterschiedliche Tests unterschiedliche Ergebnisse, dann ist der Test mit erhöhtem Ergebnis zu wiederholen

^c^Bei Vorliegen von Hyperglykämie und klassischen Symptomen ist die Diagnose ohne Testwiederholung gegeben, da z. B. bei Erstmanifestation des Typ-1-Diabetes das HbA_1c_ normal sein kann

^d^Weiterführende Diagnostik mittels Nüchternglukose oder OGTT ist erforderlich

Für die Diagnose des Gestationsdiabetes (GDM) gelten andere als die in Tab. 3 gelisteten Kriterien (s. ÖDG-Management der Hyperglykämie in der Schwangerschaft [Update 2026] für detaillierte Informationen) [13]. Die Durchführung eines 75 g-OGTT wird in der 24. bis 28. Schwangerschaftswoche bei allen Frauen ohne bereits vorbestehenden Diabetes mellitus empfohlen. Die Grenzwerte lauten nüchtern < 92 mg/dl (5,1 mmol/l), nach 1 h < 180 mg/dl (10,0 mmol/l) und nach 2 h < 153 mg/dl (8,5 mmol/l) [13]. Bereits ab einem erhöhten Plasmaglukosewert wird die Diagnose GDM gestellt. Bei erhöhtem Risiko für GDM wird jedoch eine frühere Testung vor der 20. Schwangerschaftswoche empfohlen (s. ÖDG-Leitlinien Management der Hyperglykämie in der Schwangerschaft [Update 2026] für detaillierte Informationen) [14].

Voraussetzungen zur Glukosebestimmung sind:

- ausschließlicher Einsatz qualitätsgesicherter Maßnahmen oder Tests;
- vorzugsweise Bestimmung im venösen Plasma (Zusatz von Lithium-Heparin oder besser EDTA + Natrium-Fluorid). Serumproben sind nur zu verwenden, wenn ein Glykolysehemmstoff zugesetzt wurde;
- keine Bestimmung mit Blutglukosemessgeräten, die zur Selbstkontrolle verwendet werden;
- „Nüchtern“ bedeutet eine Zeit von ≥ 8 h ohne jegliche Kalorienaufnahme;
- bei der Durchführung ist auf die mögliche Verfälschung der Diagnose durch interkurrente Erkrankungen (z. B. Infektionen, Dehydratation, gastrointestinale Krankheiten) oder Medikamenteneinnahme (z. B. Glukokortikoide) zu achten;

### HbA_1c_

Diabetes mellitus kann auch anhand der HbA_1c_-Grenzwerte ≥ 6,5 % (48 mmol/mol) diagnostiziert werden (Tab. 3). Grundlage dafür ist die Zunahme des Risikos für diabetische Retinopathie ab HbA_1c_-Werten von > 6,5 % (48 mmol/mol) [15]. Für HbA_1c_-Werte von 5,7 % (39 mmol/mol) bis einschließlich 6,4 % ist ein erhöhtes Diabetesrisiko anzunehmen, sodass in diesem Fall eine Abklärung mittels Nüchternglukosemessung und OGTT empfohlen wird. Ein starker, kontinuierlicher Zusammenhang zwischen dem HbA_1c_-Wert und der späteren Entwicklung eines Diabetes mellitus und erhöhtem kardiovaskulärem Risiko besteht [6]. Diabetes mellitus kann bei erwachsenen Personen, Kindern und Jugendlichen auch auf Basis eines erhöhten HbA_1c_-Wertes diagnostiziert werden [16]. Die Vorteile der Messung des HbA_1c_-Wertes liegen in der höheren präanalytischen Stabilität und geringerer täglicher Varianz [6]. Nachteile sind die geringere Sensitivität, höhere Kosten (somit weltweit nicht überall verfügbar) und geringere Korrelation zwischen HbA_1c_ und durchschnittlichen Blutglukosewerten. Die Bestimmung des HbA_1c_ entspricht einer indirekten Messung der durchschnittlichen Blutglukosewerte über mehrere Wochen hinweg und kann durch Einflussfaktoren wie Alter, Ethnizität und Anämie/Hämoglobinopathie von den tatsächlich gemessenen Glukosewerten abweichen [6]. Der HbA_1c_-Wert eignet sich nicht zur Diagnose von Diabetes bei Neugeborenen (HbF ca. 90 %), Schwangeren zur Diagnose von Gestationsdiabetes, Frauen bis ca. 2 Monate nach der Geburt, bei Einnahme von Blutglukose-steigernden Medikamenten, wie z. B. Glukokortikoiden, bestimmten Psychopharmaka, wenn die Einnahme < 2 Monate zurückliegt, bei Erkrankungen der Bauchspeicheldrüse einschließlich Pankreasoperationen, Bluttransfusionen, Blutspenden und starken Blutungen (Operationen, Unfälle) [7]. Von besonderer Bedeutung ist die eingeschränkte Aussagekraft des HbA_1c_-Wertes unter den folgenden Umständen (nach [7]): Faktoren, die zu einem Anstieg führen:

- Anämie, z. B. aufgrund von Eisen- oder Vitaminmangel (B_12_, Folsäure),
- Splenektomie,
- zunehmendes Alter,
- Ethnizität: z. B. höhere HbA_1c_-Werte bei Afroamerikanern und Südasiaten.

Faktoren, die zu einer Senkung führen:

- hämolytische Anämie, verursacht z. B. durch immunologische Prozesse, Medikamente wie Cephalosporine,
- Behandlung einer Eisen- oder Vitaminmangelanämie mit geeigneten Medikamenten,
- schwere Leber- oder Niereninsuffizienz,
- bei Situationen/Erkrankungen mit erhöhtem Erythrozytenumsatz (z. B.: Schwangerschaft, Hämodialyse, Bluttransfusion, großer Blutverlust, Sichelzellanämie, Thalassämie, hereditäre Sphärozytose) sollte nur die Plasmaglukosekonzentration zur Diagnose des Diabetes mellitus herangezogen werden, da der HbA_1c_-Wert in diesen Fällen falsch niedrig sein kann.

Labortechnische Probleme: Unerklärliche Abweichungen zwischen HbA_1c_ und Plasmaglukose sollten an labortechnische Diskrepanzen bei der HbA_1c_-Bestimmung denken lassen. Nähere Informationen zu Faktoren, die mit HbA_1c_-Ergebnissen interferieren können, und Assay-Interferenzen sind im Internet unter ngsp.org (http://ngsp.org/factors.asp, http://ngsp.org/interf.asp) nachzulesen. Weitere Verfälschungen von HbA_1c_ sind durch hämatologische Erkrankungen, die den Erythrozytenumsatz erhöhen (Thalassämien, pathologische Hämoglobine [HbS, HbE, HbF, HbC, HbD]) und Hämodialyse, HIV-Behandlung oder Erythropoietin-Therapie möglich.

Zur besseren Vergleichbarkeit der Methoden zur Bestimmung des HbA_1c_ sollen ausschließlich Methoden verwendet werden, die nach dem neuen Standard der International Federation of Clinical Chemistry (IFCC) referenziert sind [17, 18]. Diese Werte sollen, um Verwechslungen zu vermeiden, nach dem IFCC-Standard in mmol/mol ausgegeben werden. Die Umrechnung in den HbA_1c_-Wert in Prozent nach dem National Glycohemoglobin Standardization Program (NGSP) bzw. dem Diabetes Control and Complications Trial (DCCT) ist wie folgt:

$$\mathrm{HbA}1\mathrm{c}\,\,\text{in Prozent}=\left(0{,}09148*\mathrm{HbA}1\mathrm{c}\,\,\mathrm{in}\frac{\mathrm{mmol}}{\mathrm{mol}}\right)+2{,}152$$

Ein DCCT-HbA_1c_-Wert von 6,5 % entspricht somit einem IFCC-HbA_1c_ von 48 mmol/mol.

## Oraler Glukosetoleranztests (OGTT) nach WHO-Richtlinien

### Indikationen

Risikogruppen (s. unten), ältere Menschen (aber nicht routinemäßig), gestörte Nüchternglukose, Schwangerschaft in der 24. bis 28. Schwangerschaftswoche (s. auch: Leitlinie Management der Hyperglykämie in der Schwangerschaft [Update 2026]).

### Durchführung

- ≥ 3 Tage kohlenhydratreiche (≥ 150 g/Tag) Ernährung, 8–12 h Nahrungs- und Alkoholkarenz vor dem Test
- Testung am Morgen im Liegen/Sitzen (kein Rauchen vor/während des Tests, keine übermäßige körperliche Aktivität)
- Glukosebestimmung (Zeitpunkt 0 min)
- Trinken von 75 g Glukose (oder äquivalente Menge Stärke) in 250–300 ml Wasser (Kinder: 1,75 g/kg bis maximal 75 g Glukose) innerhalb 5 min
- Glukosebestimmung (Zeitpunkt 60 min nach Glukoseaufnahme): nur bei Abklärung von Gestationsdiabetes notwendig
- Glukosebestimmung (Zeitpunkt 120 min nach Glukoseaufnahme)

### Kontraindikationen

Interkurrente Erkrankungen, St. p. Magen-Darm-Resektion/bariatrische Operation, Resorptionsstörungen, bereits diagnostizierter Diabetes mellitus.

### Einflussfaktoren

Längeres Fasten und Kohlenhydratmangelernährung, körperliche Aktivität oder akuter Stress können auch bei Gesunden zur pathologischen Glukosetoleranz führen. Eine Reihe von Medikamenten, wie z. B. Glukokortikoide, Adrenalin (Epinephrin), Phenytoin und Furosemid, können die Glukosetoleranz verschlechtern. Auch Glykolyse ist ein oft unterschätztes Problem, wodurch Glukosekonzentrationen fälschlicherweise zu niedrig gemessen werden [19]. Bei der Blutabnahme kann ohne Zusatz von Glykolysehemmern die Glukose durch zellulären Stoffwechsel rasch abgebaut werden. Für eine präzise Bestimmung des Blutglukosespiegels sind daher die Verwendung geeigneter Abnahmeröhrchen oder die sofortige Zentrifugation und Abtrennung des Plasmas essenziell.

## Screening

Personen mit erhöhtem Diabetesrisiko sollten durch systematisches Screening auf Prädiabetes und Typ-2-Diabetes untersucht werden. Die Risikofaktoren v. a. für Typ-2-Diabetes umfassen unter anderem einen Mangel an körperlicher Aktivität und unausgewogene hyperkalorische Ernährung, die häufig die Basis für Übergewicht und Adipositas und in weiterer Folge Hyperlipidämie, arterielle Hypertonie, metabolische Dysfunktion-assoziierte steatotische Lebererkrankungen (MASLD, vormals nichtalkoholische Fettlebererkrankungen) [20] bilden (Tab. 4). Weitere Risikofaktoren stellen eine genetische Prädisposition bzw. positive Familienanamnese, eine gewisse ethnische Herkunft, zunehmendes Lebensalter sowie Sexualhormonstörungen und Gestationsdiabetes dar [21]. Zystische Fibrose und Zustand nach Transplantation von Organen sind ebenso Risikofaktoren für die Entstehung einer Hyperglykämie.

**Tab. 4:** Kriterien zur Durchführung des Diabetesscreenings bei asymptomatischen erwachsenen Personen. (Adaptiert und erweitert nach [6])

1. Ein Hyperglykämiescreening sollte bei Vorliegen folgender Risikofaktoren erfolgen:
*BMI ≥* *25* *kg/m*^*2*^* (bei asiatischer Herkunft 23* *kg/m*^*2*^*) mit einem oder mehreren der folgenden Risikofaktoren*
– Positive Familienanamnese bei erstgradigen Verwandten
– Ethnizität mit erhöhtem Diabetesrisiko (asiatische, afrikanische, lateinamerikanische Herkunft)
– Vaskuläre Erkrankungen
– Arterielle Hypertonie (≥ 130/80 mm Hg oder antihypertensive Therapie)
– HDL-Cholesterin < 35 mg/dl und/oder Triglyzeride > 250 mg/dl
– Polyzystisches Ovarsyndrom (PCOS)
– Hypogonadismus
– Körperliche Inaktivität
– Acanthosis nigricans
– MASLD*
– Chronischer Tabakkonsum**
2. Bei bekanntem Prädiabetes sollte ein jährliches Screening erfolgen
3. Bei Zustand nach GDM sollte zumindest alle 1 bis 3 Jahre ein Screening erfolgen***
4. Hochrisikogruppen (Menschen mit HIV, Exposition gegenüber risikoreichen Medikamenten, Anzeichen einer Parodontitis, Vorgeschichte einer Pankreatitis) sollten regelmäßig überwacht werden [6]
5. Bei allen anderen Personen sollte ein Screening ab einem Alter von 35 Jahren erfolgen
6. Bei unauffälligen Screeningresultaten sollte ein weiteres Screening alle 3 Jahre erfolgen. Engmaschigere Kontrollen sollten den Screeningergebnissen und Risikofaktoren entsprechend geplant werden

* Umfasst einfache Fettleber (Steatosis hepatis oder nichtalkoholische Fettleber [NAFL]), nichtalkoholische Steatohepatitis (NASH), „kryptogene“ Formen der Leberfibrose, -zirrhose und des hepatozellulären Karzinoms [20]

** Chronischer Tabakkonsum ist mit erhöhtem Risiko für Typ-2-Diabetes assoziiert [22]

*** Spezielle Risikofaktoren und Screening für GDM (s. Management der Hyperglykämie in der Schwangerschaft [Update 2026])

Vor Durchführung von Labortests empfehlen nationale Diabetesorganisationen wie die ADA oder die DDG die Durchführung von Diabetesscreeningtests zur besseren Risikobewertung (z. B.: FINDRISK, ADA Diabetes Risk Test, Deutscher Diabetesrisikotest). Bei Vorliegen einer der oben genannten Risikofaktoren sollte eine Testwiederholung in einem minimalen Intervall von 3 Jahren, bei Prädiabetes jährlich stattfinden.

Als Risikofaktor für Typ-1-Diabetes gelten Diabetes-assoziierte Antikörper, wobei das Vorliegen von 2 oder mehr dieser Autoantikörper auf ein > 80 %iges Risiko für die Entwicklung eines manifesten Typ-1-Diabetes innerhalb von 15 Jahren bei Kindern hinweist [6]. Durch Screeningprogramme für Typ-1-Diabetes für Personen mit hohem Risiko (z. B.: Familienangehörige von Menschen mit Typ-1-Diabetes) können Kinder und junge Erwachsene mit Typ-1-Diabetes oder einem Risiko dafür frühzeitig identifiziert werden [8] (s. Leitlinie Diagnostik und Therapie des Typ 1 Diabetes mellitus und Diabetes mellitus im Kindes- und Jugendalter).

Bei Verdacht auf das Vorliegen eines monogenetischen Diabetes mellitus ist eine unmittelbare molekulargenetische Testung empfohlen. Dies sollte v. a. bei Auftreten von Hyperglykämie in den ersten 6 Lebensmonaten oder bei in mehreren Generationen auftretendem Diabetes mellitus mit nicht zu Typ-1- oder Typ-2-Diabetes passenden Symptomen/Charakteristika erfolgen. Ein darauf spezialisiertes Zentrum sollte in die Versorgung dieser Personen zur Abschätzung der Signifikanz der Mutation, zur fachgerechten genetischen Beratung und Therapieplanung einbezogen werden [6]. Die Aufklärung des Betroffenen und ein genetisches Beratungsgespräch müssen entsprechend den Richtlinien des Gentechnikgesetzes erfolgen (s. auch Leitlinie „Genetische, endokrine und medikamentöse Diabetesformen“).

Bei Zustand nach Transplantation eines Organs und dementsprechend erforderlicher immunsuppressiver Therapie sowie bei zystischer Fibrose ist das Screening der Wahl ein OGTT [6]. Bei zystischer Fibrose und noch nicht diagnostiziertem zystische Fibrose-bedingtem Diabetes (CFRD) sollte der OGTT ab dem 10. Lebensjahr jährlich und bei Zustand nach Transplantation nach Stabilisierung der immunsuppressiven Therapie durchgeführt werden.

Bei asymptomatischen Kindern und Jugendlichen sollte ebenfalls ein Typ-2-Diabetes-Screening erfolgen, wenn eine Adipositas (BMI > 95. Perzentile, geschlechts- und altersadjustiert) oder ein Übergewicht (BMI > 85. Perzentile) und zusätzlich ein oder mehrere Risikofaktoren wie mütterlicher GDM in der Schwangerschaft des Kindes oder Typ-2-Diabetes bei Verwandten 1. bis 2. Grades, Hinweis auf Insulinresistenz oder mit ihr assoziierte Veränderungen oder Ethnizität mit erhöhtem Risiko vorliegen [6].

## Prävention

### Prävention des Typ-2-Diabetes

Zahlreiche prospektive Studien zur Prävention des Typ-2-Diabetes, unter anderem die Diabetes Prevention Study (DPS) und das Diabetes Prevention Program (DPP), zeigten, dass Veränderung des Lebensstils oder medikamentöse Maßnahmen eine Reduktion des Risikos der Manifestation von Typ-2-Diabetes mellitus ermöglichen (Tab. 5; [23, 24]). Mittels Lebensstilmodifikation konnte das Diabetesrisiko um 39 % und mit medikamentösen Interventionen um 36 % reduziert werden, jedoch konnte eine langfristige Risikoreduktion (28 %, durchschnittliche Beobachtungszeit 7,2 Jahre) nur mit Lebensstilveränderung beobachtet werden [23, 24]. Nach medikamentöser Intervention war keine nachhaltige Reduktion des Diabetesrisikos zu erkennen [23]. Auch die Kosten-Nutzen-Rechnung zeigt deutlich die längerfristigen positiven Effekte von Lebensstilintervention auf. In einer britischen Berechnung zur Kosteneffektivität waren sowohl Lebensstilmaßnahmen mit niedriger als auch hoher Intensität und medikamentöse Intervention mit Metformin im Vergleich zu keiner Intervention bei Anwendung an Personen mit IFG, IGT oder erhöhtem HbA_1c_ (Prädiabetes) kosteneffektiv [25]. Bislang zeigte nur die chinesische DaQing Diabetes Prevention Study eine Reduktion der kardiovaskulären und allgemeinen Mortalität bei Menschen mit IGT [26]: In der Interventionsgruppe wurde nach einem 6‑jährigen Lebensstilinterventionsprogramm die kardiovaskuläre Mortalität um 41 % (*n* = 51/430; 12 % vs. *n* = 27/138; 20 %; HR 0,59, 0,36–0,96) und die allgemeine Mortalität um 29 % (*n* = 121/430; 28 % vs. *n* = 53/138; 38 %, HR 0,71, 0,51–0,99) im Follow-up nach > 20 Jahren gesenkt. Eine Follow-up-Analyse der Da Qing Diabetes Prevention Study nach 30 Jahren zeigte eine Risikoreduktion für Typ-2-Diabetes um 39 % durch Lebensstil- und Gesundheitsverhaltensänderung [26]. Neuere Daten aus der Da Qing Diabetes Prevention Study weisen darauf hin, dass eine Regression von IGT zu normaler Glukosetoleranz oder eine Verhinderung einer Progression zu Typ-2-Diabetes nach 6 Jahren Intervention auch zu einem niedrigeren Risiko für kardiovaskuläre und mikrovaskuläre Ereignisse nach über 30 Jahren Follow-up führt [27]. Auch in der Diabetes Prevention Study wurde gezeigt, dass das Erreichen von Normoglykämie während der Intervention mit einem niedrigeren Risiko für Diabetes und mikrovaskulären Komplikationen in Zusammenhang steht [28]. Langzeitdaten des Diabetes Prevention Programs mit 21 Jahre Follow-up zeigten zwischen Metformin‑, Lebensstil- oder Plazebogruppen keine Unterschiede hinsichtlich Gesamt-, krebsbedingter oder kardiovaskulärer Mortalität [29].

**Tab. 5:** Ausgewählte Studien zur Prävention des Typ-2-Diabetes mittels Lebensstilmodifikation. Die Auswahl erfolgte nach Teilnehmerzahl (zumindest 100), Interventionsdauer (zumindest 2 Jahre) und Ethnizität (Schwerpunkt europäisch/kaukasisch) auf Basis von 2 Metaanalysen. (Nach [23, 24])

Studie	Studienarme	Teilnehmer	Beobachtungsdauer	Diabetesrisikoreduktion	Referenz
DPP	Ernährung+Bewegung, Metformin, Plazebo	3234 mit IFG oder IGT	3 Jahre	58 %	[34]
DPS	Ernährung+Bewegung, Kontrolle	552 Männer und Frauen mit IGT	Mittelwert 3,2 Jahre	58 %	[35]
Da Qing	Ernährung, Bewegung, Ernährung+Bewegung	577 Männer und Frauen mit IGT	6 Jahre	Bewegung: 37 %, Ernährung: 33 %, Ernährung+Bewegung: 32 %	[36]
IDPP	Ernährung+Bewegung, Metformin, Ernährung, Bewegung+Metformin, Kontrolle	531 Männer und Frauen mit IGT	Median 30 Monate	Ernährung+Bewegung: 28,5 %	[37]
SLIM	Ernährung+Bewegung, Kontrolle	147 Männer und Frauen mit IGT	3 Jahre	58 %	[38]
EDIPS	Ernährung+Bewegung, Kontrolle	102 Männer und Frauen mit IGT	3,1 Jahre	55 %	[39]
Zensharen	Ernährung+Bewegung, Kontrolle	641 Männer und Frauen mit IFG	3 Jahre	44 %	[40]
JDPP	Ernährung+Bewegung, Kontrolle	304 Männer und Frauen mit IGT	3 Jahre	47 %	[41]

Da sowohl Rauchen als auch Passivrauchen die Inzidenz für Diabetes erhöht, trägt eine rauchfreie Umgebung unmittelbar zur Diabetesprävention bei [30]. Ein Rauchstopp kann zwar über die mögliche Gewichtszunahme das Diabetesrisiko mittelfristig erhöhen, senkt aber gleichzeitig die erhöhte Mortalität um nahezu 50 %. Details s. Leitlinie „Rauchen, erhitzte Tabakprodukte, Alkohol und Diabetes“ [30]. Schlafmangel und schlechte Schlafqualität können zu Insulinresistenz und Glukoseerhöhung führen [31].

Bezüglich der bariatrischen (metabolischen) Chirurgie ergab eine Metaanalyse von Studien mit insgesamt fast 95.000 Betroffenen eine Diabetesremission von > 70 % [32]. Eine weitere Metaanalyse mit 39 prospektiven und retrospektiven Kohortenstudien zeigte nach bariatrischer Operation Risikoreduktionen bei kardiovaskulären Ereignissen und Mortalität (Reduktion: Myokardinfarkt 42 %, Schlaganfall 36 %, Herzinsuffizienz 50 %, kardiovaskuläre Mortalität 41 %, Gesamtmortalität 45 %) [33].

Aufgrund dieser Daten erscheint es sinnvoll, mit Personen mit erhöhtem Typ-2-Diabetes-Risiko Maßnahmen (Änderung des Essverhaltens, regelmäßige körperliche Aktivität) zu vereinbaren, die bei Übergewicht und Adipositas zu langfristiger Reduktion des Körpergewichts um 5–10 % führen (s. Leitlinie „Körperliche Aktivität und Training bei Menschen mit Diabetes [Update 2026]“). Um mehr Personen an einem Präventionsprogramm teilhaben zu lassen, sollten zusätzliche Angebote mithilfe neuer Technologien, z. B.: webbasierter, virtueller oder mobiltelefongestützter Programme, neben den traditionellen gecoachten Programmen geschaffen werden [42]. Eine rezente Metaanalyse ergab, dass durch technologiebasierte Präventionsprogramme signifikant Gewicht reduziert und auch die glykämische Kontrolle verbessert werden kann [43].

Da bereits bei Prädiabetes häufig ein erhöhtes kardiovaskuläres Risiko und Komorbiditäten des Diabetes mellitus wie Dyslipidämie oder arterielle Hypertonie vorliegen, sollten alle modifizierbaren Risikofaktoren regelmäßig kontrolliert und ggf. optimiert werden [42] (s. Leitlinien „Lipidtherapie bei Diabetes [Update 2026]“ sowie „Antihypertensive Therapie bei Diabetes mellitus [Update 2026]“).

### Ernährung

In den Lebensstilinterventionsstudien beinhaltete die ernährungsbezogene Beratung zur Gewichtsreduktion eine Reduktion der Gesamtfett- und Kalorienzufuhr [34]. Allerdings zeigen wissenschaftliche Erkenntnisse, dass es keine allgemeingültige, ideale Verteilung der Makronährstoffe – also Kohlenhydrate, Proteine und Fette – zur Prävention des Diabetes mellitus gibt [44]. Vielmehr sollte die Auswahl und Verteilung dieser Nährstoffe individuell erfolgen, basierend auf den aktuellen Ernährungsgewohnheiten, persönlichen Vorlieben sowie metabolischen Zielsetzungen [44].

Unterschiedliche Ernährungsformen wie mediterrane Ernährung oder kohlenhydratreduzierte Diäten können bei Personen mit Prädiabetes wirksam sein [42]. Pflanzenbasierte Ernährungsweisen mit geringen Mengen tierischer Produkte, vegetarische Kostformen sowie das DASH-Konzept (Dietary Approaches to Stop Hypertension) stehen laut Beobachtungsstudien mit einem verringerten Risiko für die Entwicklung eines Typ-2-Diabetes in Zusammenhang [42, 45–48].

Besondere Bedeutung kommt dabei der Gesamtqualität der verzehrten Nahrung zu. Ein höherer Score im Healthy Eating Index, im Alternative Healthy Eating Index oder im DASH-Score – jeweils Kennzahlen für gesunde Ernährungsmuster – korreliert mit einem reduzierten Diabetesrisiko [42]. Charakteristisch für diese Ernährungsformen ist ein hoher Anteil an Vollkornprodukten, Hülsenfrüchten, Nüssen, Obst und Gemüse sowie ein möglichst geringer Verzehr von raffinierten und hochverarbeiteten Lebensmitteln [42, 46] (für weitere Informationen s. Leitlinie Ernährungsempfehlungen für Menschen mit Diabetes [Update 2026]).

### Körperliche Aktivität

Regelmäßige moderate körperliche Aktivität (mindestens 30 min/Tag, 5‑mal/Woche bei moderater Intensität, 2‑mal muskelkräftigendes Training/Woche) wird bei erhöhtem Diabetesrisiko und manifestem Typ-2-Diabetes empfohlen. Durch moderate körperliche Aktivität verbessert sich die Insulinsensitivität und verringert sich das abdominale Fett. Durch Aktivitätsphasen unterbrochenes längeres Sitzen führte in Studien zu geringeren postprandialen Glukosewerten [42] (für weitere Informationen s. Leitlinie Körperliche Aktivität und Training bei Menschen mit Diabetes [Update 2026]).

### Medikamente

Bisher zeigten sich Metformin, Alpha-Glukosidasehemmer, Orlistat, Thiazolidindione (Glitazone), Insulin Glargin, SGLT2-Hemmer, „Glucagon-like Peptide 1“(GLP1)-Rezeptoragonisten, GIP/GLP1-Rezeptoragonisten und Testosteron (bei Testosteronmangel) effektiv in der Diabetesprävention [23, 24, 49, 50], wobei einige dieser Medikamente nicht mehr oder nur mehr limitiert eingesetzt werden (z. B. Alpha-Glukosidasehemmer, Orlistat, Rosiglitazon). Metaanalysen konnten eine Risikoreduktion für eine Progression des Typ-2-Diabetes mit SGLT2-Hemmern und GLP-1RAs im Vergleich zu Plazebo zeigen. SGLT2-Hemmer und GLP-1RAs waren signifikant mit einem geringeren Risiko für die Typ-2-Diabetes-Entstehung verbunden (RR 0,79; 95 %-KI 0,68–0,93 und RR 0,28, 95 %-KI 0,19–0,43) [51, 52]. In 2 Metaanalysen wurden eine Reduktion des Diabetesrisikos durch ACE-Hemmer und Sartane um ca. 25 % und eine Steigerung des Risikos durch Statine um etwa 10 % beschrieben [53, 54]. In Metaanalysen ergab sich eine moderate Reduktion des Diabetesrisikos unter Supplementation mit Vitamin D, die aber in keinem einzelnen RCT im Vergleich zu Plazebo statistisch signifikant war [55, 56]. Metformin ist das am besten untersuchte Medikament hinsichtlich Effektivität, Langzeitsicherheit und Kosteneffizienz. Bei früherem Gestationsdiabetes, Nüchternplasmaglukosekonzentrationen ≥ 110 mg/dl (≥ 6 mmol/l), HbA_1c_ ≥ 6,0 % (≥ 42 mmol/mol), Adipositas mit BMI > 35 kg/m^2^ und Alter 25 bis 59 Jahre sollte eine Verordnung von Metformin zur Senkung des Risikos der Progression von Typ-2-Diabetes überlegt werden [42]. Aufgrund der Assoziation von längerer Einnahme von Metformin mit Vitamin‑B_12_-Mangel sollte die Serumkonzentration von Vitamin B_12_ regelmäßig kontrolliert werden [42].

## Ansätze zur Prävention des Typ-1-Diabetes

Die Prävention des Typ-1-Diabetes kann auf verschiedenen Ebenen stattfinden: Maßnahmen zur Verhinderung der Entstehung einer Autoimmunität werden als Primärprävention bezeichnet, während Bemühungen, das Fortschreiten von Stadium 1 oder 2 zu Stadium 3 des Typ-1-Diabetes zu verzögern, als Sekundärprävention gelten [8]. Derzeit laufen klinische Studien mit Medikamenten, die auf folgende Mechanismen abzielen: Autoimmunreaktionen, Antigenpräsentation, Störungen der Blutglukoseregulation, Stress oder Funktionsstörung der Betazellen.

### Primäre Prävention

Etablierte Methoden sind derzeit noch nicht entwickelt, jedoch werden primäre Präventionsmethoden in Studien erprobt. Auch Impfstoffstudien in frühen Studienphasen werden durchgeführt. Enterovirusinfektionen, insbesondere CVB (Coxsackie-Virus B), stehen im Verdacht, Typ-1-Diabetes auszulösen. Impfstoffe sollen Infektionen mit bestimmten Enteroviren und somit der Entstehung eines Typ-1-Diabetes vorbeugen [57]. Neuartige Impfstoffe auf mRNA-Basis, die z. B. GAD65 codieren, sollen gezielt eine Immuntoleranz gegen Typ-1-Diabetes auslösende Autoantigene induzieren [58].

Primärpräventionsstudien bei genetisch gefährdeten Kindern setzen bisher sehr sichere – meist diätetische – Maßnahmen ein. Neuere Ansätze prüfen die orale Gabe von Insulin zur frühen Toleranzinduktion [59]. Orale Insulinimmuntherapie bei genetisch gefährdeten Kleinkindern erwies sich als sicher, zeigte jedoch keinen Effekt auf das primäre immunologische Studienziel [60]. Ein laufender RCT versucht dies nun bei Kleinkindern mit erhöhtem Risiko für Typ-1-Diabetes zu untersuchen [61]. Beobachtungsstudien wie DAISY oder TEDDY stellten fest, dass geringere körperliche Aktivität, ein höherer glykämischer Index und eine höhere Gesamtaufnahme von Kohlenhydraten mit einer rascheren Entwicklung zu klinischem Diabetes in Zusammenhang stehen [62, 63].

### Sekundäre Prävention

Einige internationale randomisierte multizentrische Studien zur Prävention von Typ-1-Diabetes waren nicht erfolgreich (z. B. DENIS, ENDIT, DIAMYD, DPT-1) [59].

In der sekundären Prävention ist Teplizumab, ein monoklonaler Antikörper gegen das T‑Zell-Oberflächenprotein CD3, die einzige von Zulassungsbehörden (FDA) genehmigte Therapie, die das Fortschreiten von Stadium 2 zu Stadium 3 des Typ-1-Diabetes verzögern kann [64]. Teplizumab zeigte bei Angehörigen von Menschen mit Typ-1-Diabetes eine Progressionsverzögerung im Vergleich zu Plazebo (mediane Zeit bis Erreichen von Stadium 3: 48,4 vs. 24,4 Monate) [64].

Mehrere Substanzen haben gezeigt, dass sie den Rückgang des C‑Peptids im Stadium 3 des Typ-1-Diabetes verzögern können, darunter: Cyclosporin, Teplizumab, Abatacept, Alefacept, Rituximab, Golimumab, niedrig dosiertes Anti-Thymozyten-Globulin, Verapamil, Imatinib und Baricitinib [8]. Die rezent publizierte PROTECT-Studie zeigt, dass Teplizumab bei Kindern und Jugendlichen mit neu diagnostiziertem Typ-1-Diabetes den Verlust der Insulinproduktion (C-Peptid) signifikant verlangsamen kann [65].

## References

[1] International Diabetes Federation. IDF Diabetes Atlas -11th edition 2025 11th https://diabetesatlas.org/media/uploads/sites/3/2025/04/IDF_Atlas_11th_Edition_2025.pdf. 35914061

[2] Federation ID. IDF Diabetes Atlas 10th edition. 2021. www.diabetesatlas.org..

[3] Schmutterer IDJ, Griebler R, Hrsg. Österreichischer Diabetesbericht. Bd. 2017. Wien: Bundesministerium für Gesundheit und Frauen; 2017.

[4] Breyer MK, Ofenheimer A, Altziebler J, Hartl S, Burghuber OC, Studnicka M, et al. Marked differences in prediabetes- and diabetes-associated comorbidities between men and women-Epidemiological results from a general population-based cohort aged 6–80 years-The LEAD (Lung, hEart, sociAl, boDy) study. Eur J Clin Invest. 2020;50(3):e13207. 31997311 10.1111/eci.13207

[5] Huang Y, Cai X, Mai W, Li M, Hu Y. Association between prediabetes and risk of cardiovascular disease and all cause mortality: systematic review and meta-analysis. BMJ. 2016;355:i5953. 27881363 10.1136/bmj.i5953 PMC5121106

[6] American Diabetes Association. 2. Diagnosis and Classification of Diabetes: Standards of Care in Diabetes-2025. Diabetes Care. 2025;48(1):S27–S49. 39651986 10.2337/dc25-S002 PMC11635041

[7] Pleus S, Tytko A, Landgraf R, Heinemann L, Werner C, Muller-Wieland D, et al. Definition, Classification, Diagnosis and Differential Diagnosis of Diabetes Mellitus: Update 2023. Exp Clin Endocrinol Diabetes. 2024;132(3):112–24. 38378016 10.1055/a-2166-6643

[8] Haller MJ, Bell KJ, Besser REJ, Casteels K, Couper JJ, Craig ME, et al. ISPAD Clinical Practice Consensus Guidelines 2024: Screening, Staging, and Strategies to Preserve Beta-Cell Function in Children and Adolescents with Type 1 Diabetes. Horm Res Paediatr. 2024;97(6):529–45. 39662065 10.1159/000543035 PMC11854978

[9] Ahlqvist E, Storm P, Karajamaki A, Martinell M, Dorkhan M, Carlsson A, et al. Novel subgroups of adult-onset diabetes and their association with outcomes: a data-driven cluster analysis of six variables. Lancet Diabetes Endocrinol. 2018;6(5):361–9. 29503172 10.1016/S2213-8587(18)30051-2

[10] Zaharia OP, Strassburger K, Strom A, Bonhof GJ, Karusheva Y, Antoniou S, et al. Risk of diabetes-associated diseases in subgroups of patients with recent-onset diabetes: a 5-year follow-up study. Lancet Diabetes Endocrinol. 2019;7(9):684–94. 31345776 10.1016/S2213-8587(19)30187-1

[11] Aly MD, Dwivedi OP, Prasad RB, Karajamaki A, Hjort R, Thangam M, et al. Genome-wide association analyses highlight etiological differences underlying newly defined subtypes of diabetes. Nat Genet. 2021;53(11):1534–42. 34737425 10.1038/s41588-021-00948-2

[12] Suzuki K, Hatzikotoulas K, Southam L, Taylor HJ, Yin X, Lorenz KM, et al. Genetic drivers of heterogeneity in type 2 diabetes pathophysiology. Nature. 2024;627(8003):347–57. 38374256 10.1038/s41586-024-07019-6 PMC10937372

[13] Kautzky-Willer A, Winhofer Y, Kiss H, Falcone V, Berger A, Lechleitner M, et al. Gestational diabetes mellitus (Update 2023). Wien Klin Wochenschr. 2023;135(1):115–28. 37101032 10.1007/s00508-023-02181-9 PMC10132924

[14] Simmons D, Immanuel J, Hague WM, Teede H, Nolan CJ, Peek MJ, et al. Treatment of Gestational Diabetes Mellitus Diagnosed Early in Pregnancy. N Engl J Med. 2023;388(23):2132–44. 37144983 10.1056/NEJMoa2214956

[15] Kowall B, Rathmann W. HbA1c for diagnosis of type 2 diabetes. Is there an optimal cut point to assess high risk of diabetes complications, and how well does the 6.5 % cutoff perform? Diabetes Metab Syndr Obes. 2013;6:477–91. 24348061 10.2147/DMSO.S39093 PMC3848642

[16] Mayer-Davis EJ, Kahkoska AR, Jefferies C, Dabelea D, Balde N, Gong CX, et al. ISPAD Clinical Practice Consensus Guidelines 2018: Definition, epidemiology, and classification of diabetes in children and adolescents. Pediatr Diabetes. 2018;19(27):7–19. 30226024 10.1111/pedi.12773 PMC7521365

[17] Colagiuri S. Glycated haemoglobin (HbA1c) for the diagnosis of diabetes mellitus—practical implications. Diabetes Res Clin Pract. 2011;93(3):312–3. 21820751 10.1016/j.diabres.2011.06.025

[18] Hübl W, Haushofer A, Weitgasser R. Gemeinsame Empfehlungen der ÖGLMKC und der ÖDG zur Referenzierung der HBA1C Bestimmung nach dem IFCC Standard. 2011. www.oeglmkc.at/down/HbA1c_Empfehlung2011.pdf.

[19] Sacks DB, Arnold M, Bakris GL, Bruns DE, Horvath AR, Lernmark A, et al. Guidelines and Recommendations for Laboratory Analysis in the Diagnosis and Management of Diabetes Mellitus. Diabetes Care. 2023;46(10):e151–e99. 37471273 10.2337/dci23-0036 PMC10516260

[20] European Association for the Study of the L, European Association for the Study of D, European Association for the Study of O. EASL-EASD-EASO Clinical Practice Guidelines on the management of metabolic dysfunction-associated steatotic liver disease (MASLD): Executive Summary. Diabetologia. 2024;67(11):2375–92. 38869512 10.1007/s00125-024-06196-3 PMC11519095

[21] Kautzky-Willer A, Leutner M, Harreiter J. Sex differences in type 2 diabetes. Diabetologia. 2023;66(6):986–1002. 36897358 10.1007/s00125-023-05891-x PMC10163139

[22] Kowall B, Rathmann W, Strassburger K, Heier M, Holle R, Thorand B, et al. Association of passive and active smoking with incident type 2 diabetes in the elderly population: the KORA S4/F5 cohort study. Eur J Epidemiol. 2010;25(6):393–402. 20369275 10.1007/s10654-010-9452-6

[23] Haw JS, Galaviz KI, Straus AN, Kowalski AJ, Magee MJ, Weber MB, et al. Long-term Sustainability of Diabetes Prevention Approaches: A Systematic Review and Meta-analysis of Randomized Clinical Trials. JAMA Intern Med. 2017;177(12):1808–17. 29114778 10.1001/jamainternmed.2017.6040 PMC5820728

[24] Glechner A, Harreiter J, Gartlehner G, Rohleder S, Kautzky A, Tuomilehto J, et al. Sex-specific differences in diabetes prevention: a systematic review and meta-analysis. Diabetologia. 2015;58(2):242–54. 25465437 10.1007/s00125-014-3439-x

[25] Roberts S, Craig D, Adler A, McPherson K, Greenhalgh T. Economic evaluation of type 2 diabetes prevention programmes: Markov model of low- and high-intensity lifestyle programmes and metformin in participants with different categories of intermediate hyperglycaemia. BMC Med. 2018;16(1):16. 29378576 10.1186/s12916-017-0984-4 PMC5798197

[26] Gong Q, Zhang P, Wang J, Ma J, An Y, Chen Y, et al. Morbidity and mortality after lifestyle intervention for people with impaired glucose tolerance: 30-year results of the Da Qing Diabetes Prevention Outcome Study. Lancet Diabetes Endocrinol. 2019;7(6):452–61. 31036503 10.1016/S2213-8587(19)30093-2 PMC8172050

[27] Chen Y, Zhang P, Wang J, Gong Q, An Y, Qian X, et al. Associations of progression to diabetes and regression to normal glucose tolerance with development of cardiovascular and microvascular disease among people with impaired glucose tolerance: a secondary analysis of the 30 year Da Qing Diabetes Prevention Outcome Study. Diabetologia. 2021;64(6):1279–87. 33608769 10.1007/s00125-021-05401-x

[28] Perreault L, Pan Q, Schroeder EB, Kalyani RR, Bray GA, Dagogo-Jack S, et al. Regression From Prediabetes to Normal Glucose Regulation and Prevalence of Microvascular Disease in the Diabetes Prevention Program Outcomes Study (DPPOS). Diabetes Care. 2019;42(9):1809–15. 31320445 10.2337/dc19-0244 PMC6702603

[29] Lee CG, Heckman-Stoddard B, Dabelea D, Gadde KM, Ehrmann D, Ford L, et al. Effect of Metformin and Lifestyle Interventions on Mortality in the Diabetes Prevention Program and Diabetes Prevention Program Outcomes Study. Diabetes Care. 2021;44(12):2775–82. 34697033 10.2337/dc21-1046 PMC8669534

[30] Brath H, Kaser S, Tatschl C, Fasching P. Smoking, alcohol and diabetes (Update 2019). Wien Klin Wochenschr. 2019;131(1):67–70. 30980165 10.1007/s00508-019-1455-z

[31] Schmid SM, Hallschmid M, Jauch-Chara K, Wilms B, Lehnert H, Born J, et al. Disturbed glucoregulatory response to food intake after moderate sleep restriction. Sleep. 2011;34(3):371–7. 21358855 10.1093/sleep/34.3.371 PMC3041714

[32] Panunzi S, De Gaetano A, Carnicelli A, Mingrone G. Predictors of remission of diabetes mellitus in severely obese individuals undergoing bariatric surgery: do BMI or procedure choice matter? A meta-analysis. Ann Surg. 2015;261(3):459–67. 25361217 10.1097/SLA.0000000000000863

[33] van Veldhuisen SL, Gorter TM, van Woerden G, de Boer RA, Rienstra M, Hazebroek EJ, et al. Bariatric surgery and cardiovascular disease: a systematic review and meta-analysis. Eur Heart J. 2022;. 10.1093/eurheartj/ehac071 PMC9123239 35243488

[34] Knowler WC, Barrett-Connor E, Fowler SE, Hamman RF, Lachin JM, Walker EA, et al. Reduction in the incidence of type 2 diabetes with lifestyle intervention or metformin. N Engl J Med. 2002;346(6):393–403. 11832527 10.1056/NEJMoa012512 PMC1370926

[35] Tuomilehto J, Lindstrom J, Eriksson JG, Valle TT, Hamalainen H, Ilanne-Parikka P, et al. Prevention of type 2 diabetes mellitus by changes in lifestyle among subjects with impaired glucose tolerance. N Engl J Med. 2001;344(18):1343–50. 11333990 10.1056/NEJM200105033441801

[36] Li G, Zhang P, Wang J, Gregg EW, Yang W, Gong Q, et al. The long-term effect of lifestyle interventions to prevent diabetes in the China Da Qing Diabetes Prevention Study: a 20-year follow-up study. Lancet. 2008;371(9626):1783–9. 18502303 10.1016/S0140-6736(08)60766-7

[37] Ramachandran A, Snehalatha C, Mary S, Mukesh B, Bhaskar AD, Vijay V, et al. The Indian Diabetes Prevention Programme shows that lifestyle modification and metformin prevent type 2 diabetes in Asian Indian subjects with impaired glucose tolerance (IDPP-1). Diabetologia. 2006;49(2):289–97. 16391903 10.1007/s00125-005-0097-z

[38] Roumen C, Corpeleijn E, Feskens EJ, Mensink M, Saris WH, Blaak EE. Impact of 3‑year lifestyle intervention on postprandial glucose metabolism: the SLIM study. Diabet Med. 2008;25(5):597–605. 18445174 10.1111/j.1464-5491.2008.02417.x

[39] Penn L, White M, Oldroyd J, Walker M, Alberti KG, Mathers JC. Prevention of type 2 diabetes in adults with impaired glucose tolerance: the European Diabetes Prevention RCT in Newcastle upon Tyne, UK. BMC Public Heal. 2009;9:342. 10.1186/1471-2458-9-342 PMC2760530 19758428

[40] Saito T, Watanabe M, Nishida J, Izumi T, Omura M, Takagi T, et al. Lifestyle modification and prevention of type 2 diabetes in overweight Japanese with impaired fasting glucose levels: a randomized controlled trial. Arch Intern Med. 2011;171(15):1352–60. 21824948 10.1001/archinternmed.2011.275

[41] Sakane N, Sato J, Tsushita K, Tsujii S, Kotani K, Tsuzaki K, et al. Prevention of type 2 diabetes in a primary healthcare setting: three-year results of lifestyle intervention in Japanese subjects with impaired glucose tolerance. BMC Public Health. 2011;11(1):40. 21235825 10.1186/1471-2458-11-40 PMC3037863

[42] American Diabetes Association. 3. Prevention or Delay of Diabetes and Associated Comorbidities: Standards of Care in Diabetes-2025. Diabetes Care. 2025;48(1):S50–S8. 39651971 10.2337/dc25-S003 PMC11635039

[43] Bian RR, Piatt GA, Sen A, Plegue MA, De Michele ML, Hafez D, et al. The Effect of Technology-Mediated Diabetes Prevention Interventions on Weight: A Meta-Analysis. J Med Internet Res. 2017;19(3):e76. 28347972 10.2196/jmir.4709 PMC5387112

[44] Evert AB, Dennison M, Gardner CD, Garvey WT, Lau KHK, MacLeod J, et al. Nutrition Therapy for Adults With Diabetes or Prediabetes: A Consensus Report. Diabetes Care. 2019;42(5):731–54. 31000505 10.2337/dci19-0014 PMC7011201

[45] Jardine MA, Kahleova H, Levin SM, Ali Z, Trapp CB, Barnard ND. Perspective: Plant-Based Eating Pattern for Type 2 Diabetes Prevention and Treatment: Efficacy, Mechanisms, and Practical Considerations. Adv Nutr. 2021;12(6):2045–55. 34113961 10.1093/advances/nmab063 PMC8634508

[46] Qian F, Liu G, Hu FB, Bhupathiraju SN, Sun Q. Association Between Plant-Based Dietary Patterns and Risk of Type 2 Diabetes: A Systematic Review and Meta-analysis. JAMA Intern Med. 2019;179(10):1335–44. 31329220 10.1001/jamainternmed.2019.2195 PMC6646993

[47] Zeraattalab-Motlagh S, Jayedi A, Shab-Bidar S. Mediterranean dietary pattern and the risk of type 2 diabetes: a systematic review and dose-response meta-analysis of prospective cohort studies. Eur J Nutr. 2022;. 10.1007/s00394-021-02761-3 35001218

[48] Lee Y, Park K. Adherence to a Vegetarian Diet and Diabetes Risk: A Systematic Review and Meta-Analysis of Observational Studies. Nutrients. 2017;9(6). 10.3390/nu9060603 PMC5490582 28613258

[49] Lundgrin EL, Hatipoglu B. Trending Modalities in Type 2 Diabetes Prevention. J Clin Endocrinol Metab. 2025;110(2):S187–S92. 39998920 10.1210/clinem/dgaf040

[50] Harreiter J, Roden M. Diabetes mellitus: definition, classification, diagnosis, screening and prevention (Update 2023). Wien Klin Wochenschr. 2023;135(1):7–17. 37101021 10.1007/s00508-022-02122-y PMC10133036

[51] Salamah HM, Marey A, Abugdida M, Abualkhair KA, Elshenawy S, Elhassan WAF, et al. Efficacy and safety of glucagon-like peptide‑1 receptor agonists on prediabetes: a systematic review and meta-analysis of randomized controlled trials. Diabetol Metab Syndr. 2024;16(1):129. 38877565 10.1186/s13098-024-01371-3 PMC11177512

[52] Mori Y, Duru OK, Tuttle KR, Fukuma S, Taura D, Harada N, et al. Sodium-Glucose Cotransporter 2 Inhibitors and New-onset Type 2 Diabetes in Adults With Prediabetes: Systematic Review and Meta-analysis of Randomized Controlled Trials. J Clin Endocrinol Metab. 2022;108(1):221–31. 36217306 10.1210/clinem/dgac591

[53] Sattar N, Preiss D, Murray HM, Welsh P, Buckley BM, de Craen AJ, et al. Statins and risk of incident diabetes: a collaborative meta-analysis of randomised statin trials. Lancet. 2010;375(9716):735–42. 20167359 10.1016/S0140-6736(09)61965-6

[54] Abuissa H, Jones PG, Marso SP, O’Keefe JH Jr.. Angiotensin-converting enzyme inhibitors or angiotensin receptor blockers for prevention of type 2 diabetes: a meta-analysis of randomized clinical trials. J Am Coll Cardiol. 2005;46(5):821–6. 16139131 10.1016/j.jacc.2005.05.051

[55] Tobias DK, Pradhan AD, Duran EK, Li C, Song Y, Buring JE, et al. Vitamin D supplementation vs. placebo and incident type 2 diabetes in an ancillary study of the randomized Vitamin D and Omega‑3 Trial. Nat Commun. 2025;16(1):3332. 40199888 10.1038/s41467-025-58721-6 PMC11978796

[56] Pittas AG, Kawahara T, Jorde R, Dawson-Hughes B, Vickery EM, Angellotti E, et al. Vitamin D and Risk for Type 2 Diabetes in People With Prediabetes : A Systematic Review and Meta-analysis of Individual Participant Data From 3 Randomized Clinical Trials. Ann Intern Med. 2023;176(3):355–63. 36745886 10.7326/M22-3018

[57] Hyoty H, Kaariainen S, Laiho JE, Comer GM, Tian W, Harkonen T, et al. Safety, tolerability and immunogenicity of PRV-101, a multivalent vaccine targeting coxsackie B viruses (CVBs) associated with type 1 diabetes: a double-blind randomised placebo-controlled Phase I trial. Diabetologia. 2024;67(5):811–21. 38369573 10.1007/s00125-024-06092-w PMC10954874

[58] Chen J, Hu Y, Chen Y, Zhou Z, Shen Y, Wang Y, et al. LNP-mRNA vaccine prevents type 1 diabetes in non-obese diabetes mice. J Control Release. 2024;375:513–23. 39278354 10.1016/j.jconrel.2024.09.020

[59] Jacobsen LM, Schatz D, Herold KC, Skyler JS. Prevention of Type 1 Diabetes. In: Lawrence JM, Casagrande SS, Herman WH, Wexler DJ, Cefalu WT, Hrsg. Diabetes in America. Bethesda (MD): National Institute of Diabetes and Digestive and Kidney Diseases (NIDDK); 2024. 38843373

[60] Assfalg R, Knoop J, Hoffman KL, Pfirrmann M, Zapardiel-Gonzalo JM, Hofelich A, et al. Oral insulin immunotherapy in children at risk for type 1 diabetes in a randomised controlled trial. Diabetologia. 2021;64(5):1079–92. 33515070 10.1007/s00125-020-05376-1 PMC8012335

[61] Ziegler AG, Achenbach P, Berner R, Casteels K, Danne T, Gundert M, et al. Oral insulin therapy for primary prevention of type 1 diabetes in infants with high genetic risk: the GPPAD-POInT (global platform for the prevention of autoimmune diabetes primary oral insulin trial) study protocol. BMJ Open. 2019;9(6):e28578. 31256036 10.1136/bmjopen-2018-028578 PMC6609035

[62] Liu X, Johnson SB, Lynch KF, Cordan K, Pate R, Butterworth MD, et al. Physical Activity and the Development of Islet Autoimmunity and Type 1 Diabetes in 5‑ to 15-Year-Old Children Followed in the TEDDY Study. Diabetes Care. 2023;46(7):1409–16. 37141102 10.2337/dc23-0036 PMC10300517

[63] Lamb MM, Frederiksen B, Seifert JA, Kroehl M, Rewers M, Norris JM. Sugar intake is associated with progression from islet autoimmunity to type 1 diabetes: the Diabetes Autoimmunity Study in the Young. Diabetologia. 2015;58(9):2027–34. 26048237 10.1007/s00125-015-3657-x PMC4529377

[64] Herold KC, Bundy BN, Long SA, Bluestone JA, DiMeglio LA, Dufort MJ, et al. An Anti-CD3 Antibody, Teplizumab, in Relatives at Risk for Type 1 Diabetes. N Engl J Med. 2019;381(7):603–13. 31180194 10.1056/NEJMoa1902226 PMC6776880

[65] Ramos EL, Dayan CM, Chatenoud L, Sumnik Z, Simmons KM, Szypowska A, et al. Teplizumab and beta-Cell Function in Newly Diagnosed Type 1 Diabetes. N Engl J Med. 2023;389(23):2151–61. 37861217 10.1056/NEJMoa2308743

